# Remodeling of the cellular membrane architecture in response to BK polyomavirus infection

**DOI:** 10.1186/s12985-026-03109-1

**Published:** 2026-02-18

**Authors:** Kateřina Bruštíková, Jitka Forstová, Barbora Holajová, Sandra Huérfano

**Affiliations:** Department of Genetics and Microbiology, Faculty of Science, BIOCEV, Vestec, Charles University, Prague, 25250 Czech Republic

**Keywords:** BKPyV, Polyomaviruses, Tubuloreticular structures, Vacuole, Autophagy, Endoplasmic reticulum, Membrane remodeling, Lipid droplets

## Abstract

BK polyomavirus (BKPyV) is a human pathogen that causes severe disease in immunocompromised individuals. Although discovered in the 1970s, important gaps in our understanding of BKPyV biology persist. Key unresolved areas include the precise molecular mechanisms governing viral latency and reactivation, the specific host and viral factors determining the virus tropism towards the urinary tract, and the intricate virus-host interactions that drive clinical pathogenesis. These unresolved biological questions have stalled the development of targeted therapeutics; as a result, no specific antiviral therapy is currently available for BKPyV-related diseases. In this review, we examine findings from both experimental models and clinical samples that investigate how BKPyV remodels host organelles and the molecular pathways underlying these alterations. We focus on BKPyV-driven changes in cellular membranes, including endoplasmic reticulum remodeling, mitochondrial disruption, the formation of endoplasmic reticulum-derived tubuloreticular structures, vacuoles, and autophagosomes, as well as the accumulation of lipid droplets. Collectively, these organelle-specific modifications highlight membrane remodeling as a central feature of BKPyV replication and pathogenesis. Addressing the key knowledge gaps in the molecular basis of virus-induced membrane remodeling will be critical for guiding the development of effective antiviral strategies.

## Introduction

More than half a century has passed since the discovery of the human BK polyomavirus (BKPyV) in the urine of a kidney transplant recipient with ureteric obstruction and graft failure [1]. Subsequent serological studies revealed that more than 80% of adults worldwide are infected with BKPyV [2–6]. Primary infection typically occurs in childhood. To date, the exact infectious route remains uncertain; however, reports suggest entry via the gastrointestinal or respiratory tracts [7–9]. In clinical settings, transmission has also been documented through blood transfusions or organ transplantation [10–12]. Following the primary infection—which is usually asymptomatic—the virus spreads to the urinary tract [8, 13]. There it establishes a persistent, lifelong infection characterized by minimal progeny production.

In immunocompetent individuals, BKPyV infection is generally asymptomatic, with only occasional virus shedding (low-level viruria). However, under conditions of immunosuppression, viral replication may be reactivated, leading to high-level viruria, the onset BKPyV-DNAemia, and histological evidence of organ involvement of varying severity. Clinically, this reactivation manifests as hemorrhagic cystitis in bone marrow transplant recipients or as BK polyomavirus-associated nephropathy (BKPyVAN) in kidney transplant patients, with graft failure occurring in more than 50% of cases [14, 15]. Increasing evidence also implicates BKPyV infection in tumorigenesis among immunocompromised hosts, particularly in bladder cancer [16, 17].

Given the absence of specific antiviral therapies and the clinical burden of BKPyV, a detailed understanding of the molecular mechanisms underlying virus-induced subcellular remodeling is critical. This review summarizes current knowledge of BKPyV biology, with emphasis on the virus-driven reorganization of host cellular organelles.

Research on BKPyV–host interactions has largely relied on in vitro systems, some of which are detailed in this review. These systems include, among others: monkey epithelial cells (Vero), human umbilical vein endothelial cells, human renal proximal tubular epithelial cells (RPTECs)—the primary targets of viral reactivation—and human microvascular endothelial cells from the bladder (HBMVECs) or the lung, which may act as viral reservoirs [18]. A smaller number of studies have also examined patient biopsy samples from BKPyV-related disease cases.

Two forms of the BKPyV viral genome exist based on the DNA sequence of the non-coding control region (NCCR): the archetype, characterized by O-P-Q-R-S blocks, and rearranged variants, which contain deletions or duplications within these blocks. Notably, in vitro studies—including those discussed in this review—primarily utilize rearranged variants, which arise from the archetype in vivo following active viral replication. While the specific NCCR architecture of the BKPyV strains in these patient cohorts remains unknown, the distinction is critical. The archetype is the standard for studying latency due to its poor replication, whereas the more efficient rearranged variants are used to mimic active infection. Although these NCCR rearrangements are thought to play a significant role in viral pathogenesis, the exact mechanisms remain poorly understood, as both rearranged and archetype sequences are detectable in BKPyV-associated nephritic kidneys [19].

### Biology of BKPyV

BKPyV is classified within the genus *Betapolyomavirus* of the *Polyomaviridae* family and is subdivided into four major subtypes (I–IV) based on genetic and serological diversity. Subtype I is the most prevalent and globally distributed [20–23]. Virions are non-enveloped with icosahedral capsids measuring 40–45 nm in diameter and possess a ~ 5 kb circular, double-stranded DNA genome. This genome is organized as a mini chromosome bound to histones (H2A, H2B, H3, and H4) and divided into two coding regions—early and late—separated by a ~ 400-bp-long NCCR. The NCCR contains the origin of replication, transcriptional promoters and multiple binding sites for cellular transcription factors, including nuclear factor I (NFI), tumor protein 53 (p53), nuclear factor kappa-light-chain-enhancer of activated B cells (NF-Κb), CCAAT/enhancer-binding protein beta (C/EBPβ), Ets-1, activator protein-1 (AP-1), specificity protein 1 (Sp1) and nuclear factor of activated T-cells (NFAT), among others [24–27].

The genome is enclosed in an icosahedral capsid composed of 72 pentamers of the major capsid viral protein 1 (VP1). Capsid stability is achieved through C-terminal interactions between pentamers, reinforced by calcium ions and disulfide bonds. Each pentamer associates with a single copy of a minor capsid viral protein 2 or 3 (VP2 or VP3), whose C-terminal region folds into a hairpin structure that interacts with VP1. In intact virions, these minor proteins are localized to the interior of the capsid. [25, 26].

BKPyV entry is facilitated by binding to cell-surface gangliosides GT1b and GD1b, which contain a 2,8-linked disialic acid recognition motif [28]. Internalization occurs via receptor-mediated endocytosis through both caveolae-dependent and independent pathways [29–31]. Furthermore, the recent description of BKPyV released in extracellular vesicles suggests that viral particles can infect cells using an alternative, receptor-independent entry pathway [32]. The virus then traffics through the endosomal sorting pathway, where passage through acidic compartments is essential for infectivity [30, 33]. Within endosomes, virions are transported along microtubules, bypassing the Golgi apparatus, and are subsequently directed to the endoplasmic reticulum (ER) with assistance from the GTPase Rab18 [30, 34–36].

While not yet demonstrated specifically for BKPyV, the related SV40 and murine polyomavirus (MPyV) utilize an additional entry pathway mediated by binding to receptors. In this model, multivalent binding to gangliosides acts as a nanoscale lipid-clustering stimulus. This stimulus directly induces deep membrane curvature and tubular invaginations, effectively bypassing the need for classical endocytic machinery during the initial stages of entry [37].

Based on studies of various polyomaviruses, including BKPyV, it is now widely accepted that within the ER, capsid rearrangements expose hydrophobic minor proteins on the viral surface. These proteins interact with the ER membrane [38–42] enabling viral translocation into the cytosol. Once in the cytosol, the C-terminal region of the minor capsid proteins—harboring a nuclear localization signal—engages the importin α/β1 pathway, directing the virus into the nucleus through nuclear pores [39]. Inside the nucleus, early and late gene expression, genome replication, and progeny assembly occur.

BKPyV encodes at least three early multifunctional proteins—large T antigen (LT, ~ 80 KDa), small T antigen (sT, ~ 20 KDa), and truncated T antigen (~ 17 KDa)—generated by alternative splicing. Additional transcripts, from the early region, have been described recently, including the so-called—’superT’ antigen and ‘MT-like T’ antigen. Nevertheless, the translation of these transcripts into proteins has not yet been confirmed. T antigens regulate early events during the viral life cycle. Specifically, they modulate host cell cycle regulators to establish an environment favorable for viral replication, facilitating viral DNA replication and activating the late gene transcription for the structural proteins VP1, VP2, VP3 and agnoprotein [25, 43, 44].

Early viral gene transcription is followed by genome replication, which is concurrent with late gene expression. This late expression yields the structural capsid proteins VP1, VP2, and VP3, as well as a small auxiliary phosphoprotein known as agnoprotein. Although its precise role remains incompletely understood, agnoprotein is implicated in genome replication, viral egress and modulation of innate immune responses [25, 45, 46]. Current evidence supports its classification as a viroporin, similar to the JC polyomavirus (JCPyV) counterpart [47].

Work on simian vacuolating virus 40 (SV40), a close relative of BKPyV, has shown that virion assembly depends on the chaperone heat shock protein 70 (Hsp70), which prevents premature capsid formation, assisted by LT as a co-chaperone [48, 49]. Viral egress is generally associated with host cell lysis [50–53]. In fact, agnoprotein is thought to facilitate the release of progeny from the nucleus. Evidence suggests that either nuclear envelope remodeling or the induction of other mechanisms of toxicity—via interactions with mitochondria and other intracellular membranes—could contribute to this process [45, 54–57]. Intriguingly, a subset of virions exits within extracellular vesicles, a phenomenon also reported for SV40 and JCPyV [32, 58, 59].

### Remodeling of the cellular membrane architecture in response to BKPyV

In mammalian cells, membrane-bound organelles include among others the ER, mitochondria, endosomes of the endo-lysosomal system, autophagosomes, lipid droplets and vacuoles. The latter two organelles are only present in response to specific signals and are described in more detail in the subsequent chapters. Membranes are central to cellular architecture, ensuring organelle integrity, mediating signaling, and coordinating trafficking and communication. Their dynamic adaptability enables rapid responses to internal and external stimuli [60, 61], thereby maintaining homeostasis.

As obligate intracellular pathogens, viruses exploit and remodel host cell membranes to complete their life cycles. Such modifications facilitate entry, membrane penetration, intracellular trafficking, genome replication, assembly, and egress. By reconfiguring membrane architecture and organelle function, viruses evade immune detection, localize host and viral factors, and establish replication-permissive microenvironments [62–67].

BKPyV-induced membrane remodeling involves alterations of the ER and mitochondria, the formation of ER-derived tubuloreticular structures (TRS), the formation of vacuoles, and autophagosomes, and the accumulation of lipid droplets. The latter have been observed to accumulate only in specific cell types, such as HBMVECs [68].

### BKPyV-induced changes in ER homeostasis and the ER architecture

The ER itself is a highly dynamic double-membrane organelle that interfaces extensively with other compartments. Continuous with the nuclear envelope, it extends into the cytoplasm as a network of tubules and cisternae. The ER orchestrates protein synthesis, folding, and modification, lipid biosynthesis, detoxification, and calcium storage [69]. Its functions are vulnerable to disruption by factors such as protein or lipid overload [70], calcium imbalance [71], oxidative stress [72], hypoxia [73], and aberrant glycosylation [74]. ER stress results in the accumulation of unfolded or misfolded proteins in the ER lumen. This activates the unfolded protein response (UPR) [75], a quality control system that suppresses global protein translation, enhances molecular chaperone production, and promotes degradation of misfolded proteins through ER-associated degradation (ERAD). ERAD relies on chaperones and transmembrane proteins to recognize misfolded proteins and channel them into retrotranslocation complexes for proteasomal degradation. If ER stress persists and homeostasis cannot be restored, the UPR initiates autophagy or apoptosis [76–79].

Viruses exploit the ER to facilitate critical steps in their life cycle, often driving extensive ER membrane remodeling [35, 80]. To reach the nucleus, where polyomaviruses replicate, they must first traverse the ER. In the case of BKPyV, structural rearrangements within the ER enable interactions with ER-resident factors, after which the virus co-opts the ERAD machinery [81]. A recent study further demonstrated activation of both ERAD and ER stress pathways, accompanied by vacuolization in tissues from BKPyV-infected patients [50].

For SV40, the virus not only requires ERAD for cytosolic entry but also induces TRS [82]. TRS have been reported in kidney biopsies from patients with BK virus nephropathy and in BKPyV-infected HBMVECs [51, 68].

Similarly, BKPyV infection of HBMVECs leads to the accumulation of lipid droplets [68], neutral lipid–storing organelles formed in the ER [83–85].

**Fig. 1:**
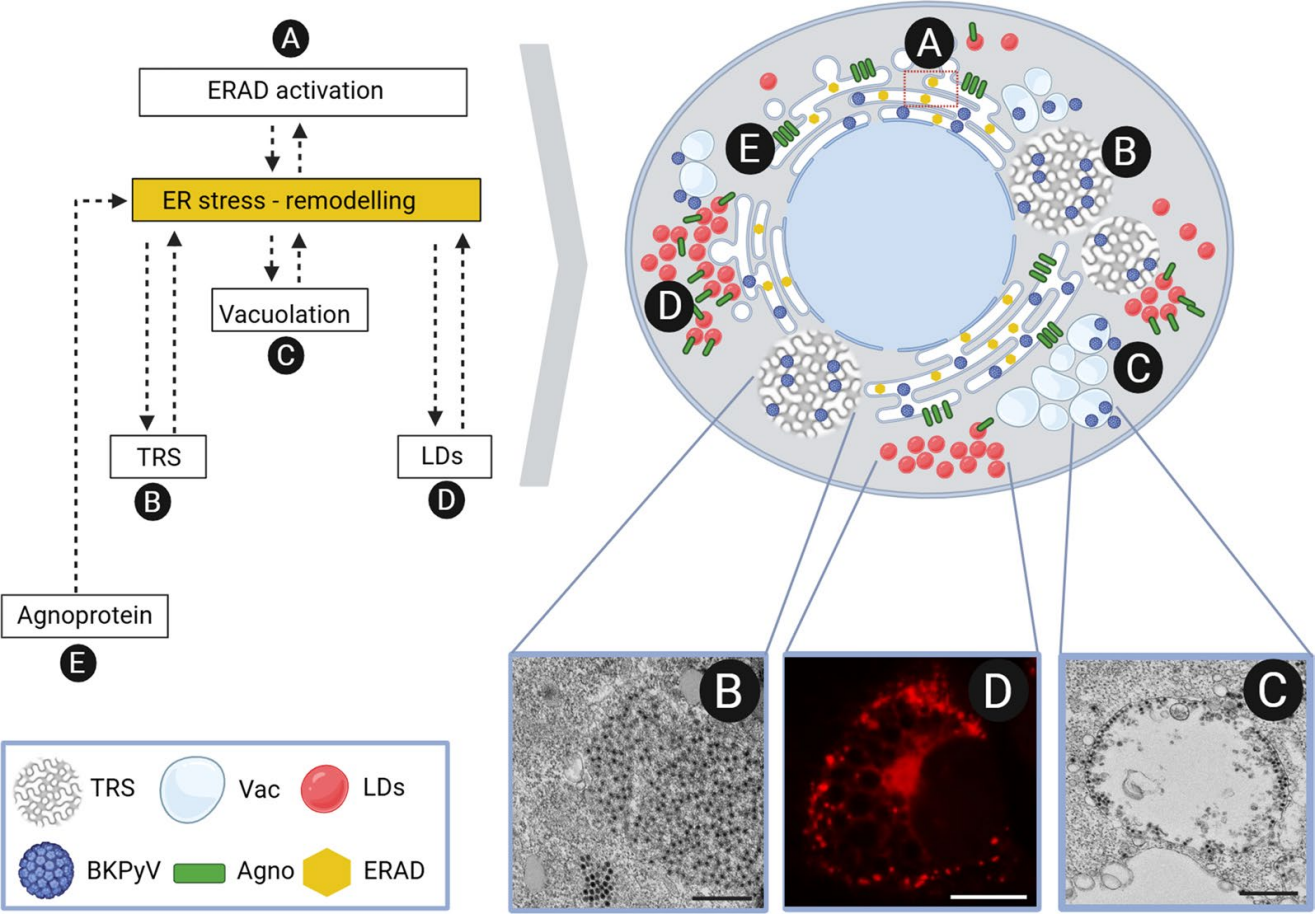
ER remodeling during BKPyV infection and its potential link to virus-induced ER stress. Left: Schematic of the interconnected processes based on findings from research articles [35, 45, 50, 51, 68, 82, 90] and syntheses from review articles [80]; top right: cartoon depiction of cellular changes; bottom right: microscopic images (scale bar B, C = 500 nm, scale bar D = 10 μm). Reprinted from BK Polyomavirus Infection of Bladder Microvascular Endothelial Cells Leads to the Activation of the cGAS-STING Pathway by Brustikova, 2024, J Med Virol (https://doi.org/10.1002/jmv.70038). CC BY 4.0. (**a**) After viral entry, BKPyV reaches the ER, where it undergoes remodeling through the ERAD pathway. Although virus activation through ER stress was suggested, the link between ERAD activation and the induction of an uncontrolled UPR leading to ER stress has not yet been elucidated. (**b**) The virus induces the formation of TRS, (**c**) vacuoles (Vac) and (**d**) lipid droplets (LDs); however, it remains unclear whether the formation of these structures is a direct result of virus trafficking–related events (endosomal sorting, ER virus–induced ER remodeling – through capsid proteins) or are a secondary consequence of ER stress triggered during infection. e) The viral protein agnoprotein (agno), expressed during the late phase of infection, binds to the ER membrane and LDs. The contribution of agnoprotein to the ER stress response is not yet understood. Created in BioRender. Bruštíková, K. (2025) https://BioRender.com/clyofao

Because ER stress is known to trigger diverse morphological and homeostatic changes—including TRS, lipid droplets, vacuoles, and autophagosomes [77, 86–89]—these structures observed during BKPyV infection may be, at least in part, induced by virus-driven ER stress. However, this connection remains to be definitively established. The viral agnoprotein, which binds and modifies intracellular membranes including the ER [45, 90], likely contributes to these ER alterations and may amplify stress responses. Figure 1 illustrates the potential interplay between virus-induced ER changes and ER stress during BKPyV infection.

### ERAD pathway activation and ER stress induction during BKPyV infection

During polyomavirus infection, capsid remodeling in the ER exposes minor capsid proteins on the virion surface. This process involves reduction and isomerization of disulfide bonds, catalyzed by ER-resident chaperones and protein disulfide isomerases. For BKPyV specifically, ER J domain-containing protein 5 (ERdj5) plays an essential role, with protein disulfide isomerase (PDI) and ER resident Protein 57 (ERp57) facilitating ERdj5-induced conformational changes, as demonstrated in SV40 studies [91, 92]. These structural changes allow the virus to engage the ERAD pathway. Indeed, BKPyV behaves as an ERAD substrate during productive infection of RPTECs, as shown by the inhibitory effects of epoxomicin (a proteasome inhibitor) and Eeyarestatin I (an ERAD inhibitor) [38]. Supporting this mechanism, ERAD translocation factors such as Derlin-1 and Derlin-2 are also required for infection by SV40, BKPyV, and MPyV [35, 92, 93].

Although ERAD activation is central to polyomavirus entry, its extent and capacity to modulate the UPR remain incompletely understood. However, a study tracking the progression of BKPyV nephropathy through public transcriptome data—using bulk RNA sequencing to compare kidney transplant patients with BKPyVAN (*n* = 28) to stable transplant recipients (*n* = 109) identified 16 ER-associated genes with differential expression. These included genes linked to the ER stress pathways such as response to ER stress, transcriptional regulation by RNA polymerase II in stress conditions, calcium signaling, calcium ion transport, and intrinsic apoptotic signaling [50]. In the same study, the authors examined five kidney biopsies from patients with BKPyVAN and two control samples; light and electron microscopy revealed that pronounced cytoplasmic vacuolization, ER expansion with partial degranulation, and abundant virions in the renal proximal tubular epithelial cells of BKPyVAN recipients, but not in controls. These findings suggest that ER stress may underlie the vacuolization observed in BKPyVAN [50].

Beyond the burden of viral entry, the viral manipulation of the cell cycle may further exacerbate organelle dysfunction. Given that BKPyV replication triggers a DNA damage response characterized by the downregulation of cyclin-dependent kinase 1 (CDK1) activity to prolong S-phase and prevent mitotic entry [94], it is plausible that the observed ER stress is, at least in part, amplified by this inhibition. The relationship between CDK1 and ER stress is well-documented and inherently reciprocal: while ER stress can trigger cell cycle arrest by inhibiting the translation of Cyclin D1 via a PERK-dependent pathway [95], specific CDK inhibitors, such as flavopiridol, are also known to actively provoke ER stress [96].

Further studies are required to demonstrate the precise level of UPR activation and its temporal regulation in BKPyV-infected cells. Investigations in RPTECs and HBMVECs, coupled with pharmacological inhibition of specific UPR pathways, may help clarify whether UPR signaling directly mediates the ER alterations induced during infection or serve to facilitate viral fitness.

### BKPyV-induced formation of tubuloreticular structures

TRS are not defined by a single marker or function but by their distinctive ultrastructural morphology. They are highly ordered, subcellular organelles derived from ER membranes, composed of interconnected, branched membranous tubules measuring 20–30 nm in diameter. These tubules form a three-dimensional reticular network within ER cisternae. TRS have been observed in diverse cell types—including endothelial cells across tissues, lymphocytes, monocytes, neural cells, and epithelial cells [97]. While they can occur in healthy cells, TRS are more commonly associated with pathological conditions such as collagen vascular and autoimmune diseases, as well as viral infections [97, 98]. Their occurrence has also been linked, in certain contexts, to elevated levels of endogenous α- and β-interferons [99].

In vitro, BKPyV viral aggregates within TRS have been documented in RPTECs three days post-infection [100] and in HBMVECs as early as 1 day post-infection [68]. Importantly, TRS have also been described in vivo in patients with BKPyV infection, underscoring their role in disease pathophysiology. Renal transplant biopsies from patients with BKPyV-associated allograft nephropathy revealed viral aggregates within TRS located near the nuclei of endothelial cells and, less frequently, epithelial cells [51]. Comparable structures have been reported in other patient biopsies, though described as multi-layered membranous formations rather than explicitly named TRS [101].

The role of TRS in host–virus interactions has been most extensively studied in SV40 infection, where they appear to facilitate viral escape from the ER into the cytosol during early infection. Super-resolution focused ion beam scanning electron microscopy revealed that virus-induced ER foci consist of tubular ER networks joined at multiple sites. SV40 preferentially localizes at these multi-tubular junctions, where the virus appears to be partially exposed to the cytosol suggesting this as a potential route for viral exit from ER to cytosol. The ER junction protein lunapark, which normally stabilizes ER branching, was shown to relocalize to these foci, supporting their structural integrity. Notably, lunapark knockdown abolishes the formation of these ER foci, consequently preventing viral escape and reducing infectivity. Confirming the requirement of lunapark in viral infection, the use of protein lunapark mutants—specifically those with disrupted zinc-finger or lunapark motifs—failed to rescue infection in knockdown cells [82].

Beyond lunapark, several proteins often implicated in reticulophagy (selective autophagy of the ER)—also participate in SV40 ER exit. These include reticulon 3 (RTN3) and reticulon 4 (RTN4), which normally stabilize the ER network [82, 102], as well as the reticulophagy receptors atlastin-2 (ATL2) and atlastin-3 (ATL3). The atlastins are dynamin-like GTPases that function as membrane-fusion machines working in close coordination with the reticulons [103]. ATL3 forms a membrane-penetration complex with lunapark, RTN3 and RTN4, and the SV40 virion through interaction with the VP1 capsid protein. ATL3 and reticulons are recruited to ER-SV40 foci to facilitate membrane curvature. While ATL2 does not relocate to these foci, it supports reticular ER morphology during virion penetration through the ER [103]. Whether BKPyV similarly exploits these proteins or induces ER-phagy during infection remains unresolved.

In addition, a recent study suggests that TRS may contribute to viral progeny release, consistent with reports of non-lytic BKPyV secretion [32, 59]. Abundant viral aggregates were observed within TRS in RPTECs at three days post-infection, even when secondary infection was blocked by neutralizing antibodies [100]. The authors proposed that TRS-associated particles may exit the cell rather than enter. This aligns with studies implicating TRS in unconventional protein secretion pathways [88].

However, it remains equally plausible that viruses are sequestered within ER-derived TRS during entry, where they may become trapped. Clarifying these possibilities will require replication-deficient viruses or virus-like particles containing minor capsid proteins capable of mimicking entry events.

### BKPyV-induced vacuole formation

Cytoplasmic vacuoles, traditionally described in plant and fungal cells as reservoirs for water, ions, and metabolites, also participate in digestion and stress responses. In animal cells, vacuoles typically emerge under stress, including bacterial or viral infection and anticancer treatment, and are often linked to forms of cell death such as paraptosis, paraptosis-like death, methuosis, or pyroptosis [89, 104]. Based on studies in plants, yeast, and protists, it is established that vacuoles primarily contain water. Consequently, in animal cells, they can also be observed via positive phase-contrast microscopy as bright inclusions or by transmission electron microscopy as single-membrane-bound vesicles with electron-lucent interiors. Such vacuolization has been documented in cells infected by BKPyV and in the kidneys of patients with BKPyVAN [50, 52, 53, 68, 105, 106]. In most cases, the direct cause of water influx into these vesicles remains unknown [107].

Vacuoles may originate from the ER or the endosomal–lysosomal system [89, 104]. The appearance of cytoplasmic vacuoles during polyomavirus infection was first described in monkey cells infected with SV40, named for this hallmark feature [108]. For SV40, vacuolization at early (4 h) and late infection stages is initiated not by viral replication but by VP1 binding to the ganglioside GM1 receptor on the cell surface. This is supported by evidence that purified VLPs and VP1 pentamers, but not GM1-binding-defective mutants or VP1 expressed alone, induce vacuolization. At late infection stages, vacuolization is massively amplified by released progeny viruses binding GM1 at the cell surface [109, 110]. Later work demonstrated that vacuolization also requires Ras–MAPK signaling, with GM1 clustering by VP1 activating Ras, which drives vacuolization, cell lysis, and virus release [111].

Vacuole formation has been documented during BKPyV infection in vitro across multiple cell types, including human embryonic fibroblasts [105], Vero cells [106], RPTECs [52, 53], and HBMVECs [68], as well as in kidney biopsies from BKPyVAN patients [50].

In RPTECs in vitro, vacuoles induced by BKPyV infection were identified as enlarged endosomal/lysosomal structures positive for early endosomal autoantigen 1 (EEA1), Ras-related protein 5 (Rab5), Ras-related protein 7 (Rab7), and the lysosome-associated membrane glycoprotein 1 (LAMP1) markers, with vacuolization occurring at both early and late stages of infection. An initial transient phase of vacuolization appeared within 3 h of infection but required high viral doses (multiplicity of infection (MOI) 100–1000) and persisted until 36 h post-infection (hpi). This process was dependent on viral binding and internalization, as neuraminidase treatment or BKPyV-specific neutralizing antibodies effectively blocked it. Interestingly, inhibition of Ras-related C3 botulinum toxin substrate 1(Rac1) had less impact, suggesting that the Ras-MAPK pathway plays a limited role in BKPyV-induced vacuolization in RPTECs. A second, late-onset wave of vacuolization, independent of viral dose, coincided with the release of progeny virions [52].

In a polarized RPTEC model infected at an MOI of 3, vacuoles were observed starting from 36 hpi. Their production increased progressively over time and was most evident at 120 hpi. Reducing the MOI resulted in a decrease in both vacuole production and other cytopathic effects. Consistent with these findings, cell toxicity became significant starting at 120 hp [53]. Similarly, in HBMVECs, infection at MOI 1–20 led to marked cytoplasmic vacuolization observed at 24 hpi, although early events were not characterized. Vacuolization intensified during later stages (3–4 days post-infection) and preceded both lactate dehydrogenase (LDH) release, indicative of cell lysis, and significant progeny production [68].

The origin and function of cytoplasmic vacuoles during BKPyV infection remain incompletely defined. However, the detection of virions on both the external and internal surfaces of vacuoles [68, 111], along with the presence of endosomal markers in some vacuoles [52], supports the hypothesis that these structures arise partly from the endosomal system. Vacuoles may thus act as intermediates in viral trafficking, directing particles either toward endosomal compartments for degradation or toward the cell periphery for progeny release. Alternatively, some vacuoles may result from ER stress, potentially contributing to virus-induced cell death.

### BKPyV-induced lipid droplet formation in human bladder microvascular endothelial cells

Recent findings further show that BKPyV infection triggers robust lipid droplet accumulation in HBMVECs, putative reservoir cells for BKPyV [68]. Lipid droplets are universal lipid storage organelles that primarily contain neutral lipids, such as triacylglycerol and sterol esters, but also harbor ~ 150 different proteins [83]. They originate from the ER, and their size, number, and composition vary with cell type and external stimuli. Beyond energy storage, lipid droplets are increasingly recognized as multifunctional organelles. They protect cells from lipotoxicity by sequestering fatty acids and contribute to homeostasis by dynamically interacting with the ER, mitochondria, peroxisomes, and lysosomes [84, 85].

Upregulated lipid droplets production is a common response to infection and cellular stress. It has been documented during autophagy triggered by prolonged nutrient deprivation [112], ER stress [87, 113], innate immune activation via Toll-like receptors (TLRs) [114, 115] or inflammasomes [116], and during proliferative signaling through Epidermal Growth Factor Receptor (EGFR) [117, 118].

In BKPyV-infected HBMVECs, lipid droplets production is abundant by 24 hpi and remains elevated through 72 hpi [68]. This contrasts with RPTECs, the primary target cells in the kidney, which display minimal lipid droplets formation during infection [45, 90]. Notably, RPTECs can generate large numbers of lipid droplets when provided with exogenous neutral lipids [45, 90], underscoring the distinct metabolic and infection-response profile of reservoir versus target cells.

Viruses can exploit lipid droplets to support their replication. For example, the dengue virus (DENV) capsid protein associates with lipid droplets, using them as scaffolds for nucleocapsid assembly during encapsidation [119]. In addition, DENV promotes lipid droplets consumption through lipophagy, releasing lipids as metabolic substrates to fuel replication and energy production [120].

Interestingly, the induction of lipid droplets and the upregulation of neutral lipid production have also been described across various other viral infections. This upregulation of neutral lipid synthesis has been documented in Zika virus, Influenza A, and Herpes Simplex Virus 1 (HSV-1) infections [117, 121], while Varicella Zoster Virus has been shown to specifically trigger the production of triglycerides [122]. Beyond mere accumulation, a clear relationship between lipid droplets and host immune responses is emerging. In this context, lipid droplets serve as immunomodulatory hubs that exert either antiviral or proviral functions, depending on the host cell type. In astrocytes, Zika virus or HSV-1 infection triggers lipid droplets production via EGFR signaling. In this context, lipid droplets are antiviral: blocking their formation with EGFR inhibitors reduces interferon levels and enhances viral replication. Conversely, increasing lipid droplets levels prior to infection boosts the interferon response and restricts the virus. Based on a broad range of experiments using viruses and agonist of DNA and RNA immune sensors, the authors concluded that although interferon is not required to induce lipid droplets, the droplets themselves are essential for boosting the interferon responses [117]. In contrast, lipid droplets play a proviral or detrimental role in dendritic cells. During HSV-1 infection, lipid droplets accumulation leads to functional impairment of the dendritic cells. Research has shown that inhibiting lipid droplets synthesis—either by blocking cholesterol esterification or fatty acid uptake—restores the ability of dendritic cells to migrate to lymph nodes in the mouse models and promotes their survival in vitro [121].

Although the role of lipid droplets in BKPyV infection remains poorly understood, evidence suggests they may contribute to viral persistence in reservoir cells such as HBMVECs. A study in RPTECs—the primary target cells of BKPyV [45]—showed that the viral agnoprotein disrupts mitochondria, impairing interferon responses. Notably, deletion of agnoprotein restored the interferon response. In addition, when lipid droplets were induced by oleate treatment, BKPyV-infected RPTECs displayed preserved mitochondrial morphology because agnoprotein was sequestered into lipid droplets. Similarly, in Vero and UTA cells, oleate treatment coupled with agnoprotein expression led to predominant agnoprotein localization in lipid droplets [45, 90]. Together, these findings support the hypothesis that the abundance of lipid droplets in reservoir cells may partially explain their preserved interferon responses during BKPyV infection.

Lipid droplets formation is strongly linked to ER stress. During stress, lipid droplets sequester excess proteins, lipids, and calcium, thereby reducing ER stress and the UPR while maintaining ER homeostasis [87]. This raises the possibility of a mechanistic link between ER stress and lipid droplets formation in BKPyV infection. Future studies could address this by treating HBMVEC-infected cells with modulators of UPR signaling and assessing their effect on lipid droplets biogenesis.

### BKPyV disrupts the mitochondrial network

Mitochondrial abnormalities have been consistently reported in RPTECs infected with BKPyV. Infected cells display small, vesicle-like mitochondria, in contrast to the elongated, filamentous mitochondrial network of uninfected cells [45].

Manzetti et al*.* [45] further demonstrated that BKPyV alters mitochondrial potential, with mutagenesis experiments identifying agnoprotein—an ER- and mitochondria-associated viral protein—as the key mediator of mitochondrial damage. Damaged mitochondria colocalized with the polyubiquitin-binding protein sequestosome 1 (p62/SQSTM1), an autophagosome marker. Silencing p62/SQSTM1 reduced microtubule-associated protein 1 light chain 3-II (LC3-II), the active autophagic marker, supporting a model in which BKPyV triggers mitophagy through agnoprotein. Importantly, similar mitochondrial alterations were detected in kidney allograft biopsies from patients with BKPyV-associated nephropathy [45].

Single-cell RNA sequencing of BKPyV-infected RPTECs revealed a gene expression pattern consistent with mitochondrial stress. Cells upregulated nuclear-encoded mitochondrial genes but downregulated mitochondrial-encoded genes. Pathway analysis confirmed mitochondrial dysfunction as the most enriched response, with reductions in oxidative phosphorylation, electron transport, mitochondrial protein import, and cristae formation [123].

Biopsy studies further highlight mitochondrial pathology in BKPyV-related disease. In an ultrastructural analysis of 10 patients with BKPyV nephropathy, mitochondria displayed severe condensation, swelling, and disruption—morphological features consistent with necrosis and apoptosis [51]. Similarly, a study of 44 patients with BKPyV-associated nephropathy and glomerular involvement reported mitochondrial swelling and degeneration in glomerular parietal epithelial cells [101]. A further study comprising five kidney biopsies from patients with BKPyVAN revealed mild swelling and focal vacuolar degeneration of mitochondria in the renal proximal tubular epithelial cells of the recipients [50].

These findings underscore the importance of mitochondria in BKPyV pathogenesis, but larger patient cohorts are needed to define the prevalence and severity of mitochondrial damage in BKPyVAN. Furthermore, in agreement with the findings in BKPyV, mitochondrial targeting by the JCPyV agnoprotein has also been described. Agnoprotein localizes to the mitochondria, where it modulates organellar metabolism. Specifically, cells expressing agnoprotein exhibit significantly reduced adenosine triphosphate production, which contrasts with the observed accumulation of calcium and the elevated production of reactive oxygen species [57]. Thus, functions of agnoprotein appear to be conserved among the human polyomaviruses.

### Autophagy processes in cells during infection by BKPyV

Autophagy is a fundamental process by which cells engulf and degrade unwanted proteins, complexes, or organelles in double-membrane autophagosomes, which subsequently fuse with lysosomes to recycle their contents, maintaining intracellular homeostasis [124]. More recently, studies have highlighted a broader role for autophagy in unconventional secretion pathways, linking it to classical exocytic processes [125].

It should be noted that the study of autophagy processes in polyomavirus infections is still in its infancy. Most importantly, controversial results have been published. BKPyV has been shown to exploit host autophagy during the early phase of infection. In Vero cells, supplementation with excess amino acids or treatment with autophagy inhibitors significantly impaired viral infection. Similarly, knockdown of the autophagy-related genes, Autophagy -related protein 7 (Atg 7) and Beclin1 reduced BKPyV infection, whereas rapamycin—an inhibitor of the mechanistic target of rapamycin (mTOR), a key negative regulator of autophagy—enhanced BKPyV infectivity. Autophagy inhibitors were most effective when administered immediately after infection, coinciding with virion trafficking to the nucleus. Consistently, interactions between virions and LC3-positive autophagosomes were detected at early post-infection time points [126]. By contrast, a study using two inhibitors of mTOR, sirolimus (SIR) and TORIN1, in RPTECs showed that both inhibited infection—an effect that was more marked when inhibition of mTOR was induced early post-infection [127]. In addition, evidence for autophagy activation during the late stages of infection is lacking. However, in JCPyV, a subset of virions released within extracellular vesicles was found to originate from secretory autophagosomes [128]. Whether a similar mechanism contributes to BKPyV progeny release remains unresolved.

As mention before, during BKPyV infection the CDK1 levels are downregulated [94], therefore it is plausible that the observed induction of autophagy is a direct consequence of this cell cycle manipulation. Indeed, CDK1 normally acts as a global inhibitor of autophagy by phosphorylating key regulatory proteins, including, among others, autophagy related protein 13 (Atg 13) and autophagy -related protein 14 (Atg 14). The suppression of CDK1 activity by BKPyV likely relieves this inhibition, thereby promoting the autophagic phenotypes observed during infection [129].

With regard to the triggers of autophagy, viral antigens appear to play a role. In RPTECs, agnoprotein expression during BKPyV infection induces mitophagy [45], as discussed earlier.

For SV40 polyomavirus, early antigens have been implicated in autophagy induction, although this has not been explored for BKPyV. A landmark 2009 study identified a novel function of the SV40 sT in cancer cells: it sustained energy homeostasis under glucose deprivation by activating AMP-activated protein kinase (AMPK). Activated AMPK in turn promoted autophagy through inhibition of mTOR, thereby providing an alternative energy source [130]. The study showed that phosphorylated AMPK levels were higher in sT expressing cells compared to controls and further increased under glucose starvation. This effect was attributed to the interaction of sT with protein phosphatase 2A (PP2A) [130, 131]. Because the BKPyV sT antigen similarly interacts with the PP2A Aα subunit [132], it is likely to exert a comparable function. Conversely, JCPyV large LT antigen has been reported to negatively regulate autophagy through its interaction with the Bag promoter and BAG protein. Bag3-induced autophagy promotes LT degradation, linking LT activity to autophagic regulation [133, 134]. Whether BKPyV early antigens mirror these roles remains to be determined.

Several fundamental questions about BKPyV-induced autophagy remain unanswered. What precisely triggers autophagy in the early stages of infection? Does ER remodeling, driven by viral translocation from the ER to the cytosol, serve as the primary stimulus? What role might BKPyV sT antigen play in sustaining autophagy during later stages of infection? Investigating subpopulations of extracellular vesicles carrying BKPyV and assessing the extent of secretory autophagy may help clarify the contribution of this pathway to viral release and dissemination. Moreover, identifying the specific extracellular vesicle subtypes generated during BKPyV infection will be essential to determine the mechanistic link between secretory autophagy and viral spread.

## Conclusions

BKPyV infection of host cells leads to subcellular changes that drive pathogenesis, although our understanding of this process is still incipient. Nevertheless, existing in vitro experiments and patient-based investigations allow us to draw some conclusions: Infection leads to the remodeling of the ER and mitochondria, and the production of autophagosomes, vacuoles, and lipid droplets. These changes are suggested to result from the virus entry process and/or be later boosted by events related to virion sorting. Furthermore, a role for a viral protein, such as agnoprotein, that may function as a viroporin in some of these events is also suggested. Last but not least, other factors, such as viral manipulation of the cell cycle or the organization of the NCCR, could influence the morphological changes induced during infection. Future research must focus on understanding, in greater detail, the molecular mechanism by which these changes are induced.

## Data Availability

Not applicable.

## Abbreviations

Agno: Agnoprotein
AMPK: AMP-activated protein kinase
AP-1: Activator protein-1
Atg 7/13/14: Autophagy related protein 7/13/14
ATL-2/3: Atlastin-2 /3
BKPyV: BK polyomavirus
BKPyVAN: BK polyomavirus-associated nephropathy
C/EBPβ: CCAAT/enhancer-binding protein beta
CDK1: Cyclin-dependent kinase 1
DENV: Dengue virus
EEA1: Early endosomal autoantigen 1
EGFR: Epidermal Growth Factor Receptor
ER: Endoplasmic reticulum
ERAD: ER-associated degradation
ERdj5: ER J domain-containing protein 5
ERp57: ER resident Protein 57
GRP78: Glucose-regulated protein 78
HBMVECs: Human bladder microvascular endothelial cells
hpi: Hours post-infection
Hsp70: Heat shock protein 70
HSV-1: Herpes Simplex Virus 1 (HSV-1)
CHOP: C/EBP homologous protein
JCPyV: JC polyomavirus
LAMP1: Lysosome-associated membrane glycoprotein 1
LC3-II: Light chain 3-II
LDH: Lactate dehydrogenase
LDs: Lipid droplets
LT: Large T antigen
MOI: Multiplicity of infection
MPyV: Murine polyomavirus
mTOR: Mechanistic target of rapamycin
NCCR: Noncoding control region
NFI: Nuclear factor I (NFI)
NF-κB: Nuclear factor kappa-light-chain-enhancer of activated B cells
p53: Tumor protein 53
PDI: Protein disulfide isomerase
PP2A: Protein phosphatase 2A
Rab 5: Ras-related protein 5
Rab 7: Ras-related protein 7
Rac 1: Ras-related C3 botulinum toxin substrate 1
RPTECs: Human renal proximal tubular epithelial cells
RTN3: Reticulon 3
RTN4: Reticulon 4
Sp1: Specificicity protein 1
SQSTM1: Sequestosome 1
sT: Small T antigen
SV40: Simian vacuolating virus 40
TLRs: Toll-like receptors
TRS: Tubuloreticular structures
UPR: Unfolded protein response
Vac: Vacuole
Vero: Monkey epithelial cells
VP1/2/3: Viral protein 1/2/3
